# Apoptotic cell-derived exosomes: messages from dying cells

**DOI:** 10.1038/s12276-019-0362-8

**Published:** 2020-01-09

**Authors:** Ramesh Kakarla, Jaehark Hur, Yeon Ji Kim, Jaeyoung Kim, Yong-Joon Chwae

**Affiliations:** 1 Department of Microbiology, Ajou University School of Medicine, Suwon, Gyeonggi-do 16499 South Korea; 2 Department of Biomedical Science, Graduate School of Ajou University, Suwon, Gyeonggi-do 16499 South Korea; 3 CK-Exogene Inc, Seoul, 54853 South Korea

**Keywords:** Apoptosis, Cell death and immune response

## Abstract

Apoptosis, a type of programmed cell death that plays a key role in both healthy and pathological conditions, releases extracellular vesicles such as apoptotic bodies and microvesicles, but exosome release due to apoptosis is not yet commonly accepted. Here, the reports demonstrating the presence of apoptotic exosomes and their roles in inflammation and immune responses are summarized, together with a general summary of apoptosis and extracellular vesicles. In conclusion, apoptosis is not just a ‘silent’ type of cell death but an active form of communication from dying cells to live cells through exosomes.

## Intercellular communication: dying cells send out messages

Dying cells communicate with healthy cells through molecular messages contained in tiny membrane-encapsulated vesicles called exosomes. In a review article, Yong-Joon Chwae from Ajou University School of Medicine in Suwon, South Korea, and colleagues discuss how these ‘apoptotic exosomes’ play important roles in immune regulation, with implications for various diseases including inflammatory disorders, cancer, neurodegeneration and infection. Researchers have long known that cells undergoing programmed cell death release vesicles that contain cellular debris, but these larger apoptotic bodies were considered mostly a messy byproduct of the dying process. Recent reports have highlighted the existence of much smaller apoptotic exosomes, each containing signals that aid in triggering and modulating immune responses. Manipulation of these exosomes or their signaling pathways could lead to new treatments for diseases linked to faulty intercellular communication and waste disposal.

## Apoptotic cell death

Apoptosis, the most commonly occurring programmed cell death process, is a highly organized and energy-dependent process involving caspases in multicellular organisms^1,2^. The apoptotic process was first described by the German scientist Carl Vogt in 1842, and the term “apoptosis” was first coined by the John Foxton Ross Kerr group in 1972^1^. Apoptosis removes many cells every single day, with adult human losses of approximately 50 billion cells on average per day^3^. In contrast to primary necrosis (traumatic cell death resulting from acute cell damage), apoptosis is a highly regulated and controlled process that aids in the removal of unwanted cells during the whole life cycle of every organism. During apoptosis, cell shrinkage and chromatin condensation occur. Due to shrinkage, the cells appear small in size with tightly packed organelles and cytoplasm. Finally, extensive plasma membrane blebbing and nuclear fragmentation lead to the formation of apoptotic bodies, which are engulfed and removed sequentially by phagocytic cells to prevent spillover and damage to the surrounding cells or tissues^4–9^.

Apoptosis can be classified into intrinsic and extrinsic pathways based on the mode of initial activation. In the intrinsic pathway (mitochondrial pathway), activation of apoptosis begins with internal signals generated from stressed cells and depends on cytoplasmic release of a protein in the mitochondrial intermembrane space, cytochrome C, through the mitochondrial outer membrane pores. BCL-2 family proteins are reported to be major regulators and effectors of mitochondrial outer membrane permeabilization, resulting in the release of cytochrome C from the mitochondrial intermembrane space^10^. BCL-2 family proteins include effector proteins (BAX and BAK), the proapoptotic BH3-only proteins (Bad, Bid, Bik, Bim, Bmf, Hrk, Noxa, and Puma) and antiapoptotic proteins (Bcl-2, Bcl-xL, Bcl-w, Mcl-1, and A1)^10,11^. BH3-only members play a key role in activating BAX and BAK directly or indirectly. Upon their activation, BAX and BAK form homo-oligomers on the outer membrane of mitochondria with formation of membrane pores, leading to cytochrome C release into the cytosol^11^. Cytosolic cytochrome C then binds to Apaf-1 and forms apoptosomes together with procaspase-9, which triggers autocleavage of procaspase-9 to active caspase-9. Activated caspase-9 further activates the caspase cascade, leading to apoptosis^4^. The activation of receptors is required for the extrinsic pathway through members of the tumor necrosis factor (TNF) receptor superfamily, including FAS (CD95) and TNF-related apoptosis inducing ligand (TRAIL) receptors. Activation of the extrinsic pathway is mediated through the binding of death ligands (TNFα, FAS, and TRAIL) to their receptors, subsequently leading to trimerization and clustering of the cytosolic death domains (DDs) of receptors, to which adapter molecules such as Fas-associated death domain (FADD) or TNFR-associated death domain (TRADD) are bound. Then, adapters recruit initiator caspases, including procaspase-8 and procaspase-10, through their death effector domains (DEDs), resulting in the formation of a death-inducing signaling complex (DISC)^4^. Then, initiator caspases activated by autocleavage in the DISC activate downstream executioner caspases, including caspase-3, caspase-6, and caspase-7^12,13^.

Caspases are a group of proteases mainly known for their crucial role in programmed cell death, including apoptosis and pyroptosis, and the inflammatory pathway. The name caspase is an abbreviation of cysteine protease activity. Caspases have been classified into apoptotic caspases, including caspase-3, caspase-6, caspase-7, caspase-8, and caspase-9 in mammals, and inflammatory caspases, including caspase-1, caspase-4, caspase-5, and caspase-12 in humans and caspase-1, caspase-11, and caspase-12 in mice^14^. In addition, based on the mechanism of action, apoptotic caspases are further classified into either initiator caspases, which include caspase-8 and caspase9, or executioner caspases, which include caspase-3, caspase-6, and caspase-7. Caspases are initially produced in an inactive form called procaspases, which require dimerization or cleavage to become active caspases^14,15^.

## Extracellular vesicles

Extracellular vesicles (EVs) are small membrane-bound vesicles that are produced from both prokaryotic and eukaryotic cells in normal physiological, as well as pathological conditions. These vesicles contain various contents, such as protein, DNA, miRNA and mRNA^16^. Based on their morphologies, modes of biogenesis, or contents, EVs are classified into three main categories: apoptotic bodies (ApoBDs), microvesicles (MVs) and exosomes^17,18^. Apoptotic bodies (ApoBDs) range from 50 to 5000 nm in diameter, close to the size of platelets, and are produced from cells undergoing programmed cell death^17,19^. During apoptosis of a cell, ApoBDs are formed during plasma membrane blebbing^9^. Later, these apoptotic bodies are phagocytosed by macrophages and fuse with lysosomes (phagolysosomes) within macrophages to prevent spillover and damage to the surrounding cell or tissue. ApoBDs can be detected using flow cytometry^20^. Microvesicles (MVs) are also known as ectosomes or microparticles^21^ and range in size from 100 to 1000 nm^22^. MVs arise by outward budding and fission of the plasma membrane. This mechanism is mediated through the interaction of phospholipid redistribution and cytoskeletal protein contraction^16^. MVs are often involved in intercellular communication, signal transduction, and immune regulation. MVs, in particular, participate in tumor invasion, metastases, coagulation, inflammation, stem-cell renewal and expansion^22^. MVs are also reported to have Annexin V, Flotillin-2, selectin, integrin, CD40, and metalloproteinase as markers^23^ and are widely detected in various biological fluids (peripheral blood, urine and ascitic fluids)^24^. Exosomes are a type of EV originating from endosomes with a size of 30–150 nm and a specific density of 1.13–1.21 g/mL. These EVs are reported to play crucial roles in intercellular communications and waste disposal in normal and pathologic conditions such as cancers, neurodegenerative diseases, cardiovascular diseases, and infectious diseases^25,26^. In particular, exosomes derived from tumors are loaded with tumor antigens that can activate dendritic cells and are involved in triggering immune responses to recognize tumors and induce cytotoxic responses against tumors. Therefore, injection of tumor exosomes can suppress tumor growth or reject tumors by inducing an immune response and subsequent activation of macrophages and natural killer cells^27^. Exosomes are produced from late endosomes called multivesicular bodies (MVBs). Invagination of the late endosomal membrane results in the generation of intraluminal vesicles (ILVs)^26^. The invagination of the endosomal membrane results in the enclosure of some proteins and cytosolic components (lipids, nucleic material including DNA, mRNA, microRNA, small-interfering RNA) within the newly formed ILVs. Finally, the fusion of MVBs with the plasma membrane releases the ILVs into the extracellular space; these molecules are then referred to as exosomes^28,29^. Exosomes can be found in most biological fluids, including urine, breast milk, plasma, saliva, cerebral spinal fluid, amniotic fluid, ascites, bile, and semen^17,30^. Exosomes have been reported to have HSP 70, CD63, CD81, CD9, LAMP1, and TSG101 as markers^23,31^.

## Apoptotic extracellular vesicles

As described above, EVs are generally classified depending on their biogenetic mechanisms as exosomes, microvesicles (MVs) and apoptotic bodies^32,33^. Exosomes and MVs have been widely studied and have important roles in several intercellular communication mechanisms, including antigen-specific immune responses mediated by exosomes with enrichment of MHC class II molecules^34–36^, and modulate anti-inflammatory effects via secretion of TGF-β1^37^. Findings reported by Valadi et al. showing that exosomes can carry nucleic acids and mRNA from mouse-derived exosomes into human cells have shed light on how EVs can contribute to intercellular communication and their potent roles in clinical application^38^. Similarly, the presence of mRNA from tumor cell-derived MVs has been reported^39^, in addition to the identification of miRNAs from the MVs isolated from blood^40^.

Similar to healthy cells, apoptotic cells can also release extracellular vesicles (termed apoptotic extracellular vesicles, ApoEVs). Among them, apoptotic bodies, which were first demonstrated by Kerr et al., were originally considered cell debris and disregarded in mainstream EV research. These molecules are generally described as vesicles with a size of up to 5 µm in diameter that carry nuclear fragments and cellular organelles such as mitochondria and endoplasmic reticulum as a result of apoptosis. Therefore, EVs for immune regulation often require the removal of apoptotic bodies because many clinical sample-derived EVs are likely to be heterogeneous^16,41,42^. Some research, however, further examined apoptotic bodies with a size of less than 1 µm and defined them as apoptotic microvesicles (ApoMVs), which are physiologically different from traditional apoptotic bodies, as they have a superior membrane integrity for molecular exchange^5,43–45^.

Apoptosis has been considered a form of ‘silent’ cell death for a long time, in contrast to necrosis, which frequently induces inflammation by releasing danger-associated molecular patterns (DAMPs). However, this notion has changed, and apoptosis has gradually been shown to participate in communication with neighboring cells to contribute to survival or apoptosis and remodeling of the surrounding tissues^46^. Some studies argue that apoptotic cell death can elicit inflammatory and immune responses under certain circumstances^47–50^, and recent studies have suggested that apoptotic vesicles derived from dying cells may be one of the main regulators of the corresponding immune regulation^41,42,51,52^. ApoEVs have been suggested to have similar characteristics to those EVs formed from healthy cells in terms of cargo delivery, including apoptotic byproducts from apoptotic clearance^42,53^, and immune regulation such as inflammation, autoimmunity, and cancer in relation to what molecular cargoes are carried^51,52,54^.

In this regard, it is worth noting that dying cells, including apoptotic cells, definitely release more EVs than healthy cells^55,56^. Moreover, a recent study showed that apoptotic cells can release MVs and exosomes in addition to ApoBDs^57^. Apoptotic MVs (0.2–1 µm in diameter), also called apoptotic microparticles, are presumed to be synthesized by plasma membrane budding in apoptotic processes and are known to induce proinflammatory cytokines through the transfer of their cargo to recipient dendritic cells or to suppress the immune system^43,58–60^.

## Apoptotic exosomes (ApoExos)

Apoptotic cell-derived exosomes or apoptotic exosomes (ApoExos) are the latest discovered entity of ApoEVs, and therefore, this name is yet generally accepted. Defining exosomes in apoptosis is difficult for several reasons. First, ApoEVs released during apoptosis are a highly heterogeneous population compared to those released from healthy cells, and thus, it is technically difficult to isolate the pure exosomal fraction^61^. Second, various EVs have common marker proteins, and thus far, unique markers for ApoExos have not been well studied^33,44^. Third, it is fairly difficult to determine that the exosomal fraction from apoptosis originates from endosomes because dying cells rapidly go through phenotypic changes from cellular shrinkage and plasma membrane blebs to either disintegrated cell bodies, called apoptotic bodies, or pyroptic changes called secondary necrosis^62,63^.

Nevertheless, the presence of ApoExos was elucidated in the serially published seminal articles of Hebert’s group. These researchers reported the caspase 3-dependent formation of MVBs and the release of ApoExos in endothelial cells, resulting in the delivery of Translationally Controlled Tumor Protein (TCTP); inhibited apoptosis of vascular smooth muscle cells; and the active 20S proteasome core in circulating ApoExos, which induced production of anti-perlecan autoantibodies and allogeneic graft rejection. These results confirmed that ApoExos contribute to intercellular communication and immune responses, similar to exosomes from healthy cells^64–66^. In addition, in glioblastoma multiforme (GBM), an aggressive brain cancer, it was recently reported that components of the spliceosomes contained in ApoExos promote tumor cell proliferation and confer therapeutic resistance to live tumor cells via remodeling of the RNA splicing patterns^67^. Interestingly, ApoExos produced from endothelial cells deliver noncoding RNAs, characterized by sequences rich in uridine and reminiscent of viral RNAs, which can stimulate RIG-I-like receptors and TLRs to induce inflammation^68^, while ApoExos from thymocytes suppresses immune responses by induction of TGFβ in macrophages^69^.

Recent research has further identified ApoExos, which share common features of exosomes with regard to their physical characteristics, such as size, density and protein expression, in addition to their roles in intercellular communication^52^. Proteins such as CD63, CD9, CD81, and HSP70 are widely recognized as marker proteins of^21,32^ exosomes, and it is generally agreed that the biogenesis of exosomes is highly associated with the endosomal-lysosomal pathway accompanied by the ESCRT complex^70^. ApoExos, as shown by Park et al., express the typical exosomal marker CD63 in addition to the lysosomal marker LAMP1 and the stress-associated protein HSP70, which is expressed under apoptotic conditions. These apoptotic vesicles have been termed apoptotic exosome-like vesicles (AEVs) with unique protein markers (i.e., Sphingosine 1-Phosphate Receptors 1 and 3, S1PR1/3) and have been proposed to be induced by damage associated molecular patterns (DAMPs). These changes induce proinflammatory cytokines, including IL-1β, from macrophages through S1P/S1PR signals in ApoExos and consequent activation of NF-κB and p38 MAPK in macrophages. Biogenesis of ApoExos has been reported to be associated with S1P signaling initiated by S1PR1/3 from the plasma membrane at the early apoptotic phase, progressing to endosomal maturation, mainly by the downstream G_βγ_ subunit of S1PR1/3 and subsequent actin mobilization^52^.

Despite the limited information on ApoExos, the data described above clearly show that ApoExos could be associated with various human pathophysiological conditions as either DAMPs or carriers for functional molecules to modulate recipient cells, which are produced by a unique MVB maturation pathway with wholly different cargos from conventional exosomes^52,71^. Thus, exosomes from dying cells are neither simple remnants nor disintegrated cell bodies that needed to be scavenged but are instead final messages from dying cells to the remaining live cells.

## Concluding remarks

**Fig. 1:**
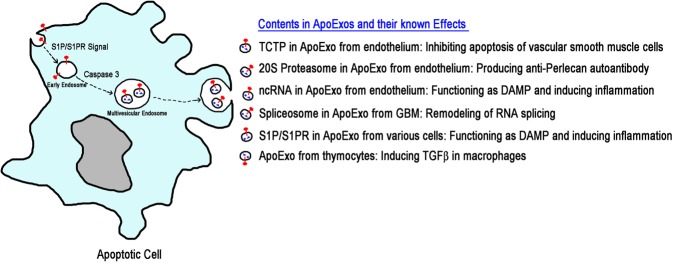
Illustration depicting current findings on ApoExos. Biogenesis of ApoExos begins with S1P/S1PR signals on the plasma membrane and requires caspase 3 for maturation of MVBs. ApoExos are associated with various pathophysiologic events, such as vascular homeostasis, autoimmunity and the resultant graft rejection, sterile inflammation, and proliferation and survival of tumors.

ApoExos exist and play a crucial role in various pathological and physiological conditions of humans despite the limited known information, as summarized together with the biogenetic mechanisms in Fig. 1. Thus, future studies have revealed the roles of ApoExos in various disease settings. These works should focus on human diseases whose etiologies are implicated in chronic inflammation or immune responses, such as cancers, chronic allergies, autoimmune diseases, and neuroskeletal and musculoskeletal degenerative diseases, given that most reports elucidated the roles of ApoExos in the induction of inflammation or specific immune responses as described above. In another aspect, ApoEVs are extremely heterogeneous with relatively high levels; thus, purification and definition of ApoExos, intermingled with other types of ApoEVs, must be carefully approached^9^.

The level of ApoExo release is completely different in the various types of cells^52^, suggesting that only the cells or tissues equipped with the systems necessary for ApoExo biogenesis could release ApoExos; otherwise, specific cellular stresses leading to apoptotic cell death might be needed for ApoExo release. However, unfortunately, the biogenetic mechanisms of ApoExos are poorly understood and have only begun to be elucidated. Hence, efforts to investigate the biogenetic mechanisms and functional studies should be undertaken. These studies would provide helpful clues for the development of new therapeutics based on modulation of ApoExo release in human diseases and promote future use of these molecules as vehicles of drug delivery and gene therapy similar to exosomes^72–75^.
